# A qualitative systematic review of the impact of hearing on quality of life

**DOI:** 10.1007/s11136-024-03851-5

**Published:** 2024-11-23

**Authors:** Nadine Henderson, Sian Hodgson, Brendan Mulhern, Katie Page, Chris Sampson

**Affiliations:** 1 The Office of Health Economics, London, UK; 2 University of Technology Sydney, Sydney, Australia

**Keywords:** Hearing disorders, Health-related quality of life, Qualitative research, Patient reported outcome measures

## Abstract

**Purpose:**

Hearing loss, deafness, and other hearing-related conditions can significantly impact quality of life; numerous qualitative studies have sought to describe these impacts. Synthesis of these findings may provide additional or more robust insights.

**Methods:**

A qualitative systematic review of studies reporting qualitative data relating to the impact of hearing problems on adults’ health-related quality of life. A subset of studies was included in the review and subsequently analysed using a thematic approach.

**Results:**

The literature search yielded 129 studies, of which 22 met our inclusion criteria and were included for analysis. The included studies, primarily from Australia, the UK, and the USA, involved approximately 450 participants with various hearing conditions. Semi-structured interviews and focus groups were the most common data collection methods, with thematic analysis being the predominant analytical approach. Three overarching categories of descriptive themes were identified: Physical, Mental, and Social. Physical encompassed sound localization, sound clarity, speech, and physical fatigue. Social included relationships, isolation, communication, independence, work function, social stigma, and confidence. Mental encompassed depression, anxiety, listening effort, mental fatigue, fear, and identity. The identified themes shed light on the diverse domains of health-related quality of life affected by hearing conditions.

**Conclusion:**

Differences in hearing function impact upon people’s health-related quality of life in a variety of ways relating to physical, mental, and social aspects of health, and these themes are clearly demonstrated across qualitative studies. These results will inform the development of hearing-specific questionnaire items for with the EQ-5D descriptive system, a commonly used patient-reported outcome measure.

**Supplementary Information:**

The online version contains supplementary material available at 10.1007/s11136-024-03851-5.

## Abstract (plain language summary)

Hearing loss, deafness, and other hearing-related conditions can significantly affect a person’s quality of life and well-being. Previous research has investigated in what way and to what extent hearing problems have an impact on adults’ health-related quality of life, which is a concept routinely used to inform the allocation of resources in health care. This study reviews and analyses the findings of previous research to identify aspects of quality of life that are important to people living with hearing conditions. We found that the affected areas of quality of life can be categorized into Physical, Mental, and Social aspects. Physical comprised of sound localization, sound clarity, speech, and physical fatigue. Social included relationships, isolation, communication, independence, work function, social stigma, and confidence. Mental comprised of depression, anxiety, listening effort, mental fatigue, fear, and identity. These findings will inform the development of hearing-specific questionnaire items to describe people’s health-related quality of life. It’s important to use qualitative research to inform the development of patient-reported outcome measures to ensure that areas that are most important to patients are included using wording that is understandable by the target participants.

**Supplementary Information:**

The online version contains supplementary material available at 10.1007/s11136-024-03851-5.

## Introduction

The EQ-5D has been validated in many clinical areas [1]. However, in some circumstances, the EQ-5D descriptive system is not sensitive to all of the health impacts of certain conditions or interventions, including hearing conditions [2–5]. Recent research has shown that members of the general public perceive the EQ-5D descriptive system to be missing important aspects of health, including sensory deprivation [6]. The EQ-5D has also been found to lack construct validity in hearing, meaning that the benefits of binaural hearing may not be captured consistently [7]. Previous exploratory research has indicated that an additional survey item (or items) referred to as a ‘bolt-on’ can improve the measurement properties of the EQ-5D [8–10].

The development of patient-reported outcome measures (PROMs), including bolt-ons, should involve qualitative research with people who have lived experience of the relevant health conditions being measured or described [11–13]. This is to ensure that measures capture aspects of health that are relevant and framed in a way that is meaningful to people completing them. For many health conditions, there is a vast literature using qualitative research to understand how health status influences quality of life (QoL). This is the case in many hearing conditions across a variety of settings [14–16].

Qualitative synthesis involves the collection, comparison and analysis of previous research findings on a topic to identify important and recurring themes [17–19]. Building on previous research allows for findings from a diverse range of studies with different research focuses to be brought together to generate new themes. Qualitative synthesis has been used to identify the domains of hearing loss to inform frameworks for patient reporting that recognize the perspectives of adults with hearing loss and their communication partners [20]. Previous research has also used qualitative review with a focus on QoL to inform the development of a health and well-being PROM [21]. To the best of our knowledge, this is the first qualitative systematic review focusing on the impact of hearing conditions on quality of life.

The primary objective of this paper is to inform the development of a hearing bolt-on for the EQ-5D by building on existing knowledge from published qualitative research. The secondary objective is to consider the themes identified in the qualitative systematic review alongside existing approaches to the description of (hearing-related) health states.

## Methodology

Qualitative synthesis involves the analysis of previously published qualitative data [17, 18]. In this research, we use qualitative synthesis to understand the impact of hearing conditions on health-related quality of life (HRQoL). Throughout this paper, we use the phrase ‘hearing conditions’ as a catch-all term for deafness, hard of hearing, tinnitus, hearing disorders, or any other cause of hearing impairment that might impact a person’s QoL. The inclusion criteria and abstract screening tool were documented in a study protocol, which was approved by the wider project team before the commencement of the screening process (available from corresponding author on request). The protocol was informed by previous qualitative syntheses and published guidance [22].

### Literature identification

We searched PsycInfo, PubMed, and Embase. Search terms included: (a) Qualitative Research OR Focus Groups OR Interview AND (b) Hearing Loss OR Hearing Disorders OR Deafness OR Tinnitus OR Hearing Aid OR Cochlear Implant OR Meniere’s Disease AND (c) Quality of life OR Happiness OR Personal Satisfaction.

Inclusion criteria are specified in Table 1. The search strategy was modified to accommodate the settings of each database and, where possible, was limited to adult participants, English language, and post-January 2000. The literature review start date was specified to capture the experiences of people living with hearing conditions in recent years. We limited our search to studies including participants over 18 years of age, as the EQ-5D is designed to measure the QoL of adults. Our full PubMed search strategy can be found in Supplementary Material 2. The search was conducted in August 2021. An update to the literature search for the period August 2021 to August 2024 can be found in Supplementary Material 1.

**Table 1:** Inclusion criteria

Participants	People with personal experience of hearing problems
	People over the age of 18 years
Study design	Reported primary data collection
	Used a recognized qualitative data collection methodology
	Stated purpose of assessing the impact of hearing problems on health-related quality of life (or closely related concepts)
Reporting characteristics	Published in the year 2000 or later
	Published in the English language
	Published as a full paper or study report

Articles not written in English were excluded because the research team did not have the resources to support translation. No exclusion criteria were specified regarding the countries where data collection occurred. There were no restrictions by setting, causes of hearing problems, relevant interventions, or type of qualitative analysis.

### Study selection

Following deduplication, three stages of study selection were conducted: {1} title screening, {2} abstract screening, and {3} full-text screening. All articles were screened by one researcher (NH), who recorded exclusion decisions and reasoning. At each stage of the screening process, another researcher (CS) reviewed the decisions. Any disagreements were resolved by discussion, and where there were any uncertainties, articles were retained for review.

During the first stage of the study selection, studies that could not satisfy the inclusion criteria (Table 2) based on the title were excluded. Examples of reasons for exclusion were participants were children, hearing impairment was not the primary condition, non-patient perspective (clinician or spouses), or study focus was not primarily QoL. Next, the abstracts of articles were screened by NH using the abstract screening tool in Table 2. All six criteria needed to be satisfied in the affirmative to be selected for inclusion. During stage 3 of the study selection, the full text of studies was screened by NH,

**Table 2:** Screening tool for abstract screening

Q1. Does the abstract contain the use of a primary qualitative data collection method?
Q2. Was the study published in or after 2000?
Q3. Is the abstract available in English?
Q4. Is hearing loss, deafness or other hearing condition the primary condition of interest?
Q5. Does the study sample include participants 18 years old and over?
Q6. Does the study include participants who have personal experience of hearing impairment?

### Data extraction

NH extracted verbatim quotes, themes, and findings related to the objective of our research during stage 3 of the study selection. For example, in studies that collected data from both clinicians and individuals with hearing impairment, clinicians’ quotes were not extracted. Study characteristics were extracted by SH and verified by NH. The following study characteristics were extracted from the included articles: publication date, countries in which data were collected, sample size, method of data collection, type of analysis, and research focus.

### Synthesis of results

The purpose of our qualitative synthesis is to understand the most important ways in which hearing conditions affect people’s QoL. Verbatim quotations from study participants, and themes and findings supported by the study data, were analyzed in NVivo version 10, 2012. These articles and their characteristics are shown in Table 3. We conducted a thematic synthesis of the included studies [23]. This involved line-by-line extraction of data and the organization of identified codes into descriptive themes describing how hearing problems affect people’s QoL. These descriptive themes were allowed to arise organically from the literature without restriction according to their appropriateness in the context of an EQ-5D bolt-on. We compared the descriptive themes to the five domains of the EQ-5D. Condition-specific PROMs are available in the context of hearing including the International Classification of Functioning, Disability and Health (ICF) Hearing Core Set [24]. The development of this was supported by the World Health Organization and informed by input from adults with hearing loss. Given the comprehensive development of this instrument, we chose to compare the themes identified from the qualitative synthesis with the ICF Hearing Core Set. We compared the descriptive themes to the 27 domains of the Brief ICF Core Set for Hearing Loss [24].

**Table 3:** Summary of the 22 articles included in the qualitative synthesis

Reference (author, year)	Country/countries of data collection	Participant sample (type of hearing problem)	No. of participants	No. of data points, e.g., focus groups, interviews)	Method of data collection	Type of analysis	Research focus (e.g., patient groups or outcomes of interest)
Barlow et al. [44]	UK	Late’ deafness - deafness in adulthood (Including physical disturbance such as tinnitus and balance disorders)	8	Each participant was interviewed once individually	Semi-structured interviews	Framework analysis	The experiences of adults with late deafness (experiences of those who had experienced the hearing world)
Bennion et al. [40]	UK	Age-related hearing impairment	9	Each participant was interviewed once individually	Semi-structured interviews	Descriptive thematic analysis	Issues with the promotion of hearing aid use and the improvement of rehabilitation services for older people
Brooker et al. [35]	Australia	Acoustic Neuroma	21	4 focus groups (Group sizes: 4, 5, 5, 7). Each participant attended 1 focus group	Focus Groups	Thematic analysis	Acoustic neuroma patients’ perceptions of their quality of life
Buhagiar & Lutman [45]	UK	Patients who got 2 cochlear implants sequentially	NR	‘Outcomes from Bilateral Cochlear Implantation (Adults)’ questionnaire given to 25 participants to answer twice. No participant numbers given for the open-ended questionnaire or face-to-face interviews	Retrospective open-ended questionnaire, face-to-face interviews and close-ended questionnaire	Grounded Theory	Differences in quality of life after having each cochlear implant
Davis et al. [34]	USA	Varying degrees of hearing loss	43	8 Focus Groups (Focus group sized ranged from 3–5 (median 5)	Focus Groups	Thematic analysis	Listening-related fatigue
Dixon et al. [36]	No specific countries reported. Patients were English-speaking	Hearing loss patients with a broad range of hearing loss causes, configurations, and severities	34	1 Focus group (8 clinical experts) Semi-structured individual interviews (26 adults with hearing loss)	Systematic Literature review, Semi-structured interviews, and focus groups	Thematic analysis	Quality of life associated with hearing loss
Duchesne et al. [33]	Canada	Congenital or prelingual deafness with a cochlear implant	21	21 participants completed the initial questionnaire. 7 of these participants were interviewed a year later	Questionnaire (adapted into an interview format) and semi-structured interviews	NR	Benefits to patients with congenital or prelingual deafness from receiving a cochlear implant as an adult
Hughes et al. [46]	UK	Severe-profound sensorineural hearing loss (SNHL) and either eligible for Cochlear implant or had a Cochlear Implant	17	3 Focus groups (group sizes (SNLH individuals): 4,5, 4) and pilot focus group containing 2 SNHL individuals. In addition to the SNLH individuals, 2 significant others with self-reported normal hearing participated in the focus groups with their partner	Focus Groups	Grounded theory method	Listening effort
Ingram et al. [43]	USA	Hearing loss	20	Each participant was interviewed once individually	Interviews and Focus Groups	Thematic analysis	Quality of life with hearing loss and disparities in access to hearing health care
Jeffs et al. [39]	UK	Congenitally or early profoundly deafened candidates who receive cochlear implants as adults	8	Each participant was interviewed once individually	Semi-structured interviews	Grounded theory method	Experiences of cochlear implants
Kushalnagar et al. [41]	NR	Congenital/Early Deafness	19	Each participant was interviewed once individually	Semi-structured interviews	Thematic analysis	Quality of life of adults with congenital or early deafness
Lucas et al. [37]	UK	Asymmetric hearing loss (single-sided deafness)	8	3 group interviews (group sizes: 2, 2, 4)	Group Interviews	Thematic Analysis	Longer term psychological and social consequences of single-sided deafness
McAbee et al. [53]	NR	Deaf	6	All 6 participated in initial structured interviews. 4 of these individuals participated in follow-up interviews	Structured Interviews	Thematic Analysis	Quality of life of deaf individuals
McRackan et al. [38]	USA	Cochlear Implant users	43	3 focus groups (group sizes: 4, 9, 10). 20 different interview participants	Focus groups and cognitive interviews	Thematic Analysis	Quality of life of individuals with cochlear implant to aid development of a Cochlear implant quality of life instrument
Mealings et al. [42]	Survey Participants: Australia, US and UK Interviews: Australia	Normal audiogram or mild hearing loss (NA-MHL)	21	233 NA-HML and 47 clinicians answered the exploratory survey. 21 NA-MHL and 7 clinicians interviewed	Exploratory survey and empathy interviews	Content analysis	Understanding the experiences of people with NA-MHL (with a focus on difficulties in listening to noise)
Ng et al. [51]	NR	Cochlear Implant users	8	8 interviews, 149 questionnaire respondents	Online questionnaire (open and closed questions) and semi-structured interviews	Grounded theory approach	Quality of life of cochlear implant users
Powell et al. [52]	USA	Hearing loss	40	Each participant was interviewed once individually	Semi-structured interviews	Thematic analysis	Hearing loss in rural population
Pryce & Chilvers [56]	England	Tinnitus	13	Each participant was interviewed once individually	Semi-structured interviews	Grounded theory method	Experiences of tinnitus patients
Punch et al. [54]	USA	Bilateral sensorineural hearing loss	8	1 focus group containing all 8 participants	Focus Group	Content analysis	Hearing-related quality of life
Rapport et al. [48]	Australia, UK	Older adults with severe SNHL	55	5 Focus groups, 8 interviews, 54 demographic questionnaires and 46 qualitative surveys	Focus groups, individual interviews, demographic questionnaires, open-ended survey	Thematic analysis	Hearing healthcare experiences (including the perspective of both patients and healthcare professionals)
Vieira et al. [32]	Brazil	Cochlear Implant users	16	Each participant was interviewed once individually	Semi-structured interviews	Grounded Theory	Changes in quality of life from cochlear implant users
Yuan et al. [47]	China	Sudden Sensorineural Hearing Loss (SSNHL)	23	Each participant was interviewed once individually	Semi-structured interviews	Colaizzi’s seven-step phenomenological analysis	Understanding the experiences (coping strategies, psychological state and needs) of SSNHL patients

NR: Not Reported

We used the Critical Appraisal Skills Programme (CASP) Qualitative Studies checklist [25] to assess the quality of included studies because it is the most commonly used checklist for quality appraisal in health and social care-related qualitative syntheses [26, 27]. We used an optimized version of the CASP tool, which provides additional considerations and structure when assessing the quality of qualitative research [28]. The results of the CASP quality assessment are included in Supplementary Material 3.

## Results

**Fig. 1:**
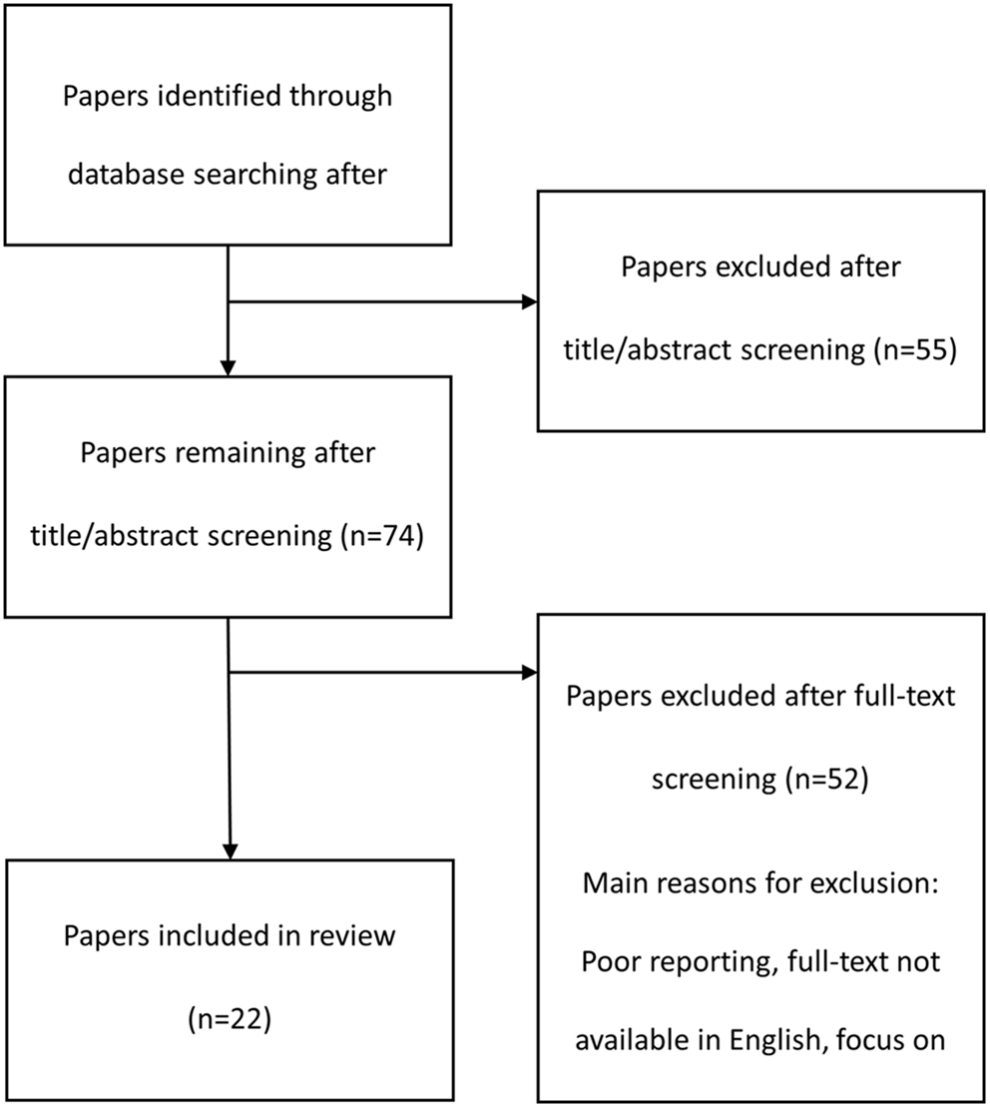
Qualitative literature search results

In the title screening phase, in two instances it was not possible to establish whether participants were over 18 years old as they were referred to as “young people” or “adolescents” [29, 30]. These articles were retained so that the age of the sample could be checked in the abstract/full text. If the study included participants both over and under 18 years old, the study was excluded. In the abstract screening phase, there were two instances of uncertainty. In the first instance, it was difficult to conclude whether primary qualitative data collection had occurred; the full text was retained for screening [31]. In the second instance, the study focus was reported as understanding the benefits of cochlear implants (CI), which includes the impact on QoL; the full text was retained for screening [32]. Twenty-two papers were included in the qualitative synthesis; the full selection process and outcomes are shown in Fig. 1.

The included papers had a combined sample size of approximately 450 participants (uncertain due to reporting) with a variety of hearing conditions, including congenital or early profound deafness, sudden sensorineural hearing loss, age-related hearing impairment, and tinnitus. Seven studies focused on participants who had undergone interventions such as cochlear implantation or the provision of hearing aids. Two papers focused on mild hearing loss.

Data collection covered six countries: Australia, Brazil, Canada, China, the UK, and the USA. The most common countries were Australia, the UK, and the USA. Most studies collected qualitative data from a single country (*n* = 16/22), and the largest proportion of those were in the UK or England (*n* = 7/22).

Studies employed semi-structured interviews (*n* = 13/22), focus groups (*n* = 5/22) or both (*n* = 4/22) as data collection methods; some used supplementary questionnaires (*n* = 6/22). The most common type of analysis employed was thematic analysis (*n* = 11/22); other methods used were grounded theory (*n* = 6/22), framework approach (*n* = 1/22), content analysis (*n* = 2/22), and Colaizzi’s seven-step phenomenological analysis (*n* = 1/22). One study did not report the type of analysis used [33].

### Synthesis of results

**Fig. 2:**
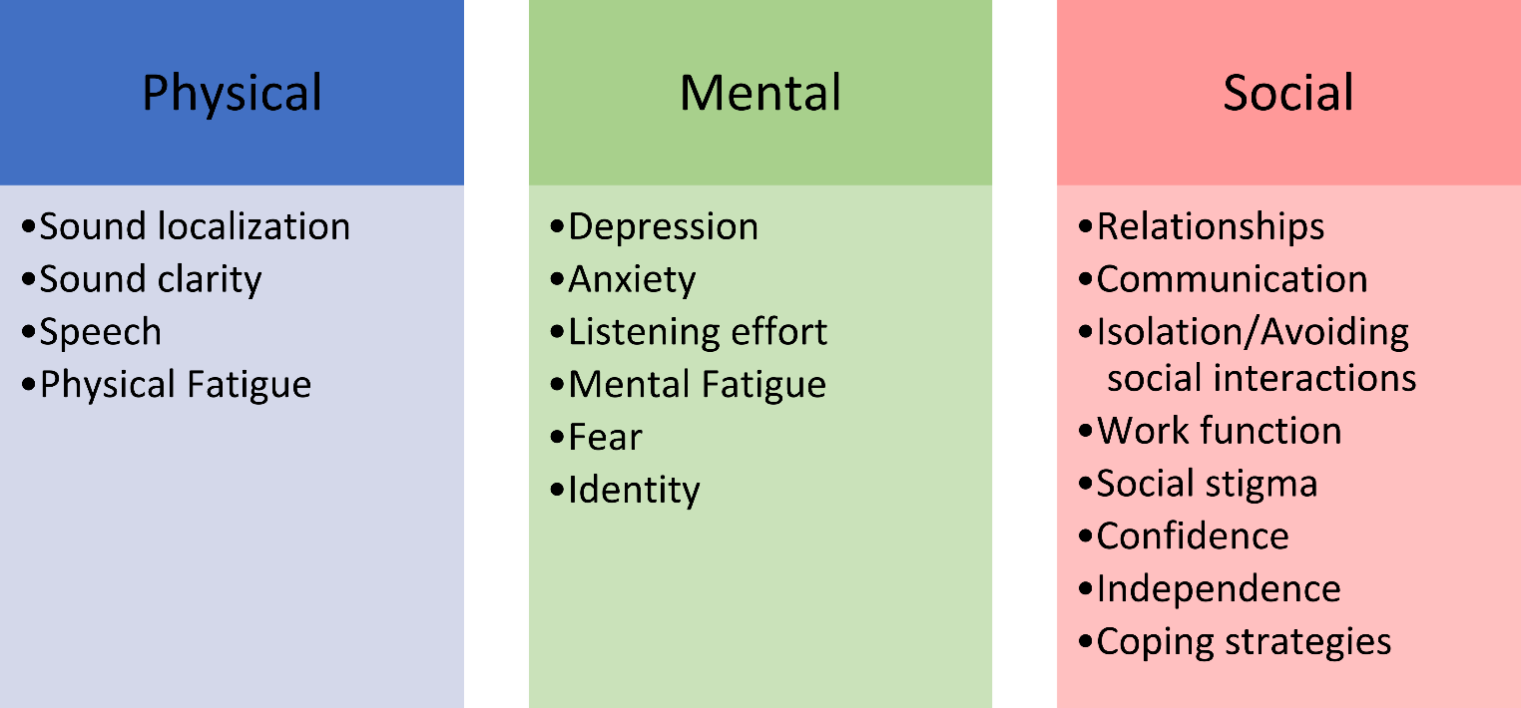
Descriptive themes

We identified numerous descriptive themes. Here, we summarize them within three categories corresponding to widely-recognized domains of health and well-being: physical, mental, and social. Some themes are multi-faceted; we allocate them based on the principal or primary impact, as described by authors of the included studies (Fig. 2).

#### Physical

Within the physical category, we included physical symptoms, such as fatigue; sensory experiences, such as sound clarity; and the need for physical or behavioural changes associated with hearing problems, including selective attention, physical repositioning, and production of speech. Davis et al. [34] characterize the physical manifestations of listening-related fatigue, stating that “reports of exhaustion, low energy, sluggishness, and tiredness, were common among participants” and that “participants agreed that demanding listening could be physically draining”.

##### Sound localization

Participants reported difficulties with sound localization and the ability to selectively attend to one sound, which necessitates the need for some participants to position themselves on their better-hearing side, sometimes causing physical discomfort [32, 35–37]. An example of this is noted by Dixon et al. [36]: “People would call my name, I’d actually do a 360 to see where people are calling from… you really don’t have any idea”.

##### Sound clarity

Cochlear implant users reported improvements in sound clarity post-intervention, suggesting that they had previously had difficulties with this aspect of hearing [32, 38].

##### Speech

Many participants report that understanding speech is a challenge, especially in noisy environments [36–39]. Depending on the level of hearing impairment, some participants report that they have a heavy reliance on lip reading [37, 39, 40]. Cochlear implant users reported that their implants facilitated their intelligibility and speech production, thereby improving their understanding by others [32, 39]. This is captured by this individual’s experience:

When I was deaf, as I couldn’t hear well, my speech was quite tangled. People had difficulty understanding me. After the implantation, I could hear the sound of my voice better, and because of that, my speech began to get better, clearer, and more understandable [32].

##### Physical Fatigue

Individuals with hearing impairment frequently report physical fatigue; this is associated with physical repositioning to help in social situations [34, 35, 37, 41, 42]. For those with asymmetric hearing impairment, turning their heads to ensure that the source sound is on their non-impaired side is often tiring and uncomfortable [37].

#### Mental

Within the mental category, we included affective functioning, including behavioural, subjective, emotional, and motivational aspects. Examples include depression, anxiety, fear, and avoidance. Ingram et al. [43] reported that participants with hearing loss described “negative emotions in response to hearing loss, including depression, sadness, desperation, frustration, shame, and embarrassment”.

##### Depression

In some studies, authors reported depression and low self-esteem associated with hearing loss [35, 44, 45]. This was mainly reported by participants who had suffered from sudden hearing loss [38, 44].

##### Anxiety

Anxiety was linked by some participants to their perception of the difficulty and effort required in upcoming listening situations [34, 35, 37, 42, 46, 47]. Anxiety was also mentioned by participants in the context of worrying about employment and providing for families [44].

##### Listening effort

Hearing impairment often requires participants to exert additional effort to understand others [34, 37, 38, 42, 46, 48]. This was often associated with both mental and physical fatigue [34, 37, 38, 41]. For a full systematic review of the effect of hearing impairment and hearing aid amplification on listening effort, see Ohlenforst et al. [49].

##### Mental fatigue

Several participants reported mental fatigue from making adjustments as a result of their hearing impairment [34, 37, 41]. One individual recalled the fatigue associated with the extra listening effort in a classroom setting:

I think the mental fatigue comes from not only trying to keep up [with what is being said in class] and just do the basics, but it’s also from all these extra things we have to do as people with hearing impairments … You suddenly realise I’ve spent so much energy on all these little things that no one else spends it on [34]!.

For a full discussion of the effect of hearing loss and hearing device fitting on fatigue (in general), see Holman et al. [50].

##### Fear

Participants who experienced sudden hearing loss sometimes reported feelings of fear and shock [37, 38, 46, 51]. Some individuals reported fearing for their safety when crossing roads or not being able to hear fire alarms [37, 40, 52].

##### Identity

Some individuals reported hearing impairment brought about changes to their self-identity; some stated that they felt less sure of themselves and their self-confidence had declined [48]. Others felt less sure of their identity after hearing loss because they felt they did not fit in with the “hearing world” or “prelingually deaf world” [39, 44].

#### Social

Within social, we included a broader construct that includes themes affecting interpersonal relationships, such as work, family, communication, and self-concepts (confidence, stigma, independence). Barlow et al. [44] summarize the impact of acquired hearing loss on participants’ identity and social well-being, conveying that they felt that the onset of deafness had “left them between worlds, in a twilight zone, and had robbed them of their identity”.

##### Relationships

Many of the included papers identified ‘Relationships’ as a key theme relating to the QoL of people with hearing impairment [32, 36, 43–45, 48, 52, 53]. Participants reported that hearing loss negatively impacted existing relationships and that they find developing new social relationships challenging [36, 43, 52].

##### Communication

The ability to communicate has a significant impact on the QoL of those with a hearing impairment; this was a common theme across the included papers [32, 33, 36–38, 41–44, 46–48, 52, 54]. As described in the Relationships theme, communicating with family and friends was frequently mentioned by participants; however, some also reported that communicating with clinicians, work colleagues, or people over the telephone posed difficulties [44, 52, 53].

##### Isolation/avoiding social interactions

The feeling of isolation was frequently reported by participants, with some actively avoiding social gatherings to avoid embarrassment and fatigue [32, 34, 35, 37, 38, 40, 43–46, 51, 53]. Feelings of isolation were often associated with group conversations and/or noisy environments [34–37, 46]. These sentiments are captured in this quote: “I feel a sense of that isolation that you’re not part of the group any longer, because you can’t actually hear what’s going on. You tend to withdraw, well I do anyway, because I really can’t follow what’s going on” [35]. Others reported that their friends and acquaintances avoided having conversations after the onset of hearing loss, resulting in feeling ignored and misunderstood [38, 43, 44]. For a full discussion of hearing loss and social isolation, see Shukla et al. [55]

##### Work function

Many participants mentioned work function or employment in the context of hearing loss [32, 35, 36, 38, 41, 44, 47, 51, 54, 56]. Some participants reported that they were no longer able to work in their previous jobs, chose to retire early because of hearing loss or that having an implant had allowed them to regain employment or find their first job [32, 38, 44, 54]. Other participants reported difficulties in maintaining their ability to perform their responsibilities due to problems communicating with colleagues and a lack of support from employers [36, 41, 54]. For a full discussion of the association of hearing loss and employment, see Shan [57].

##### Social stigma

Some studies reported that social stigma negatively impacted QoL for those with hearing impairment, stemming from a lack of understanding or empathy from others [37, 54]. Some individuals reported often being wrongly perceived as being rude or antisocial if they failed to respond to someone calling their name or saying, “Excuse me” [37, 54].

##### Confidence

In studies focusing on participants who had received cochlear implants, an increase in personal confidence was reported, and an increase in their families’ confidence in the participant’s abilities [32, 33, 38, 39, 46, 48, 51]. In studies focusing on participants who had experienced sudden or gradual hearing loss, including older adults, a loss of confidence was a common theme [44, 52]. One study cited that confidence and the ability to be independent are central to participants who consider themselves part of the Deaf community [53].

##### Independence

Independence was a key theme across papers, with CI users reporting increased independence [32, 38, 45, 51] and Deaf people valued their independence in the context of QoL [53].

##### Coping strategies

Many individuals mentioned the use of coping strategies; this often included informing communication partners of the need to see their faces when they speak to facilitate lip-reading, sitting in certain places to hear better, and situating themselves to avoid background noises [40, 41, 54, 56]. One individual recalled how they accommodated for their hearing when inviting friends to social gatherings: “Whenever we had people come to our house we generally only invited one other couple and I insisted that they sit around the table to talk to me” [35].

#### Comparison to EQ-5D items and brief ICF core set for hearing loss

We compared the themes identified in our synthesis with the brief International Classification of Functioning (ICF) Core Set for hearing loss to ascertain the face validity of our themes and to provide an understanding of the comprehensiveness of our review. We conceptually mapped themes to the 27 domains of the brief ICF Core Set using the descriptive labels of each domain and the ICF definitions to assist in interpretation where differences were simply due to terminology (e.g., ‘occupational impacts’ versus ‘remunerative employment’). This comparison is shown in Table 4.

**Table 4:** Conceptual mapping between our identified themes and EQ-5D and the ICF Core Set for hearing loss

	Qualitative synthesis themes	EQ-5D	ICF core set
Physical	Sound Localization	*No match*	Localization of Sound Source
	Sound Clarity	*No match*	Sound Discrimination - Sensory functions relating to sensing the presence of sound involving the differentiation of ground and binaural synthesis, separation and blending.
	Speech	*No match*	Speech discrimination - Sensory functions relating to determining spoken language and distinguishing it from other sounds.
	*No match*	*No match*	Vestibular functions - Sensory functions of the inner ear related to position, balance and movement.
	*No match*	*No match*	Sensations associated with hearing and vestibular functions - Sensations of dizziness, falling, tinnitus and vertigo.
	Physical Fatigue	*No match*	*No match*
Social	Relationships	*Usual Activities (partial match)*	Informal social relationships
			Family relationships
			Intimate relationships
	Isolation/Avoiding social interactions	*No match*	*No match*
	Communication	*No match*	Conversation
			Using communication devices and techniques
	Independence	*No match*	*No match*
	Work function	Usual activities	Remunerative Employment
			School education
			Higher education
	Confidence	*No match*	Temperament and personality function
	Coping strategies	*No match*	No match
Mental	Depression	Anxiety/Depression	*Emotional functions (partial match)*
	Anxiety	Anxiety/Depression	*Emotional functions (partial match)*
	Listening Effort	*No match*	*Attention functions (partial match)*
	Mental Fatigue	*No match*	*No match*
	Fear	*No match*	Emotional functions
	Identity	*No match*	*Temperament and personality function*

Half of our themes (8 of 16) could be mapped to a domain from the ICF Core Set. Themes that could not be matched to the ICF Core Set included those in the Mental category as well as ‘physical fatigue’, ‘isolation’, and ‘independence’. Two notable domains from the ICF Set that were not closely related to our descriptive themes were ‘vestibular functions’ and ‘sensations’ associated with hearing and vestibular functions.

The identified themes were broader than those covered by commonly used generic QoL measures, including the EQ-5D. The descriptive themes ‘depression’ and ‘anxiety’ are captured by the EQ-5D, but other descriptive themes (in the Physical and Social categories) may not be captured by the EQ-5D. It’s likely that the themes within our Social category collectively contribute to an individual’s ability to perform their usual activities, but the research team agreed that there are too many other aspects outside of social well-being for the individual descriptive themes to match with the Usual Activities domain.

Our synthesis indicated that making adjustments to day-to-day life to cope with hearing loss, from physical repositioning and having social interactions in a quiet environment to the use of hearing aids and cochlear implants, are common among people with hearing problems, further substantiating previous research [58, 59]. Within the context of the EQ-5D, coping strategies are important for people with hearing impairments to be able to perform their usual activities. There is evidence to suggest that there is a disparity between the valuation of the impact of hypothetical conditions of health and the reported health by those experiencing them; this discrepancy may be partly explained by adaptation to living with a health condition over time [60].

### Quality assessment

The CASP checklist was applied to the 22 qualitative papers included in this synthesis. Overall, the reporting of the studies was adequately detailed, and relevant considerations were undertaken. An area in which studies tended to be reported poorly was risk of bias resulting from the researcher conducting the qualitative work; with 11 of 22 studies reporting this adequately. Data analysis reporting was generally adequate, with 16 of 22 studies describing the analysis process in detail.

### Mapping connections between themes

**Fig. 3:**
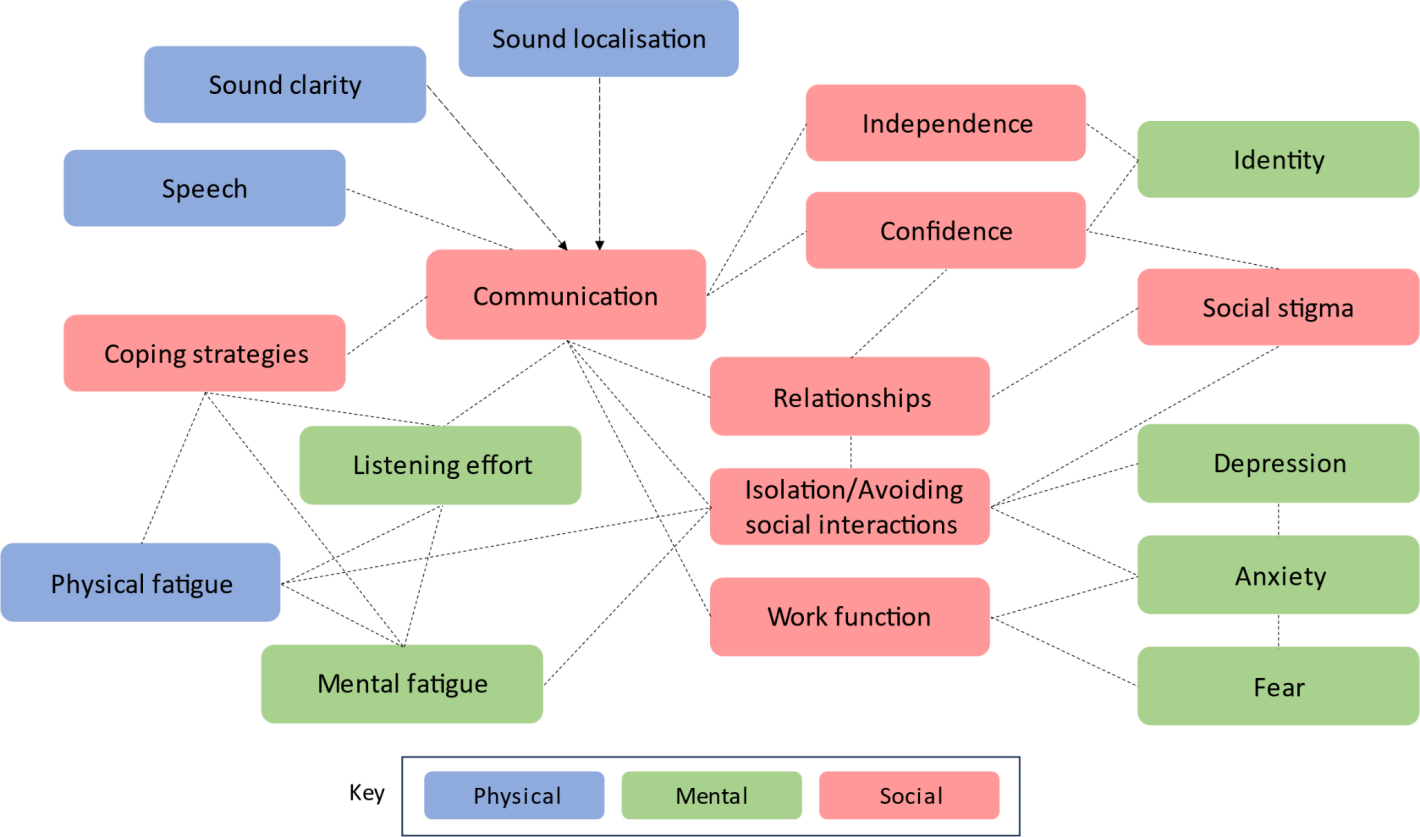
Mapping of connection between themes

Many of the themes are associated with other themes within and across categories. We visualized potential associations between themes in Fig. 3, where arrows denote single-directional impacts, and all other lines denote bidirectional associations. The relationships arose from our synthesis of the literature and are supported by findings or verbatim quotes from at least one or more papers; note that the map is not theory-driven and was not informed by previous work in this area.

The main implications are that many aspects that impact the QoL of people with hearing loss are interconnected and affect communication. An example of knock-on impacts is that participants reported increased listening effort as a result of hearing loss, which often led to fatigue, which could lead to anxiety and avoidance of social interactions. The use of coping strategies was challenging to categorize; overall, the team agreed that coping strategies were mainly necessitated in social situations but that certain coping strategies could impact or be impacted by aspects of physical or mental health.

## Discussion

Hearing conditions can have a wide-ranging impact on individuals’ quality of life, we identified numerous important themes across three domains. Some of these themes were strongly related, particularly in the Social and Mental categories, as discussed above. There is some degree of interconnectedness between the themes, with some themes having spillover or knock-on effects on other themes, indicating the complexity of factors impacting on the QoL of people with hearing conditions. Indeed, the downstream impacts of hearing loss have been linked to depression and cognitive decline in later life [61, 62].

In terms of the overlap between our themes and the EQ-5D domains, we observe that some important themes relevant to hearing-related QoL are not directly captured by the EQ-5D domains, further substantiating previous work [6, 63]. It’s unclear whether people with hearing conditions would consider themes included in our Social category, such as ‘communication’ and ‘relationships’, as part of the Usual Activities domain. The themes included in the Social category may align more closely with some of the domains included in the EQ-HWB, such as ‘autonomy’ and ‘relationships’ [64].

### Differences across hearing conditions

There may be differences in the type and extent of the impact on QoL depending on the specific hearing condition and severity and how they are reported by individuals. Two key factors identified in our results may contribute to these differences: the onset of hearing loss (i.e., congenital/prelingual hearing loss or deafness vs. acquired hearing loss) and the use of hearing assistance devices (cochlear implants or hearing aids). Furthermore, the severity of hearing loss and other physical symptoms, such as those associated with tinnitus, are likely to contribute to an individual’s QoL to varying degrees (Table 5). Many studies included individuals from different groups, making it difficult to make reliable comparisons. For example, McRackan et al. [38], Ng et al. [51], and Vieira et al. [32] focus on the QoL of people with cochlear implants, which includes groups C and D from Table 5. Whereas Kushalnagar et al. [41] and Duchesne et al. [33] focus on people with congenital or early deafness, including groups A and C. Therefore, an area for further research could be to investigate trends in how QoL is reported across these (or similar) groups.

**Table 5:** Potential categorization of study participants

	Congenital or prelingual hearing loss	Acquired hearing loss
No cochlear implant or hearing aid	A	B
Has a cochlear implant or hearing aid	C	D

### Limitations

Overall, the included papers covered a wide range of severity of hearing conditions, which is crucial for informing the development of generic measures of HRQoL for use in the context of a variety of conditions and severity. However, only one paper included focused on the experiences of people living with tinnitus. The literature search identified some additional studies focusing on tinnitus and Meniere’s disease, which were excluded based on the primary qualitative data collection criterion. This may have led to some physical symptoms being missed by our qualitative synthesis, for example, symptoms related to sensations or dizziness as described in the ICF core set (Table 4). Similarly, we may not have captured research with a specific diagnostic focus. For example, since the literature search date, relevant qualitative studies have explored the impact of hearing loss on the QoL of adults with multi-drug resistant tuberculosis and chemotherapy-induced ear damage on cancer survivors [65, 66].

The literature search was conducted in August 2021; an update to the original search to August 2024 can be found in Supplementary Material 1. The themes in the update broadly align with the themes identified in this article, with additional themes identified in the update: access to medical/social services and financial issues.

Research has shown that over 80% of the population with hearing loss lives in low- and middle-income countries (LMICs), where access to relevant health care may be limited [67]. The majority of studies included in our synthesis were conducted in high-income countries, meaning that we are unlikely to have captured the perspectives of a large proportion of people living with hearing loss, particularly untreated hearing loss. This limitation may have been partly caused by our English language restriction or a lack of qualitative research on the impact of hearing on QoL conducted in LMICs; further research is needed in this area. Since the literature search date, research has been published exploring the QoL and challenges faced by people with hearing loss in Cameroon [68].

## Conclusion

Hearing conditions have a considerable impact on numerous domains of (health-related) quality of life, both hearing condition specific domains and more generally, which can be categorized into three main areas: Physical, Mental and Social. Through our mapping process, we observe that some of our descriptive themes are not fully captured by the EQ-5D, especially those specific to hearing conditions. Our findings will inform the specification of candidate items and corresponding descriptors for a hearing bolt-on, including further qualitative and quantitative testing. Qualitative synthesis is a valuable tool for informing item development by ensuring maximum pre-existing information is considered in the development process.

## Electronic Supplementary Material

Below is the link to the electronic supplementary material.

Supplementary Material 1

## Data Availability

Not applicable.
