# Supplementary material for: A qualitative systematic review of the impact of hearing on quality of life
Source: Qual Life Res. 2024 Nov 23;34(4):879–92. doi: 10.1007/s11136-024-03851-5 (PMC11982117; doi:10.1007/s11136-024-03851-5)
Supplement: Supplementary file 1 — Supplementary Material 1 [file 11136_2024_3851_MOESM1_ESM.docx]

A Qualitative Systematic Review of the Impact of Hearing on Quality of Life: Supplementary Material

# Supplementary Material 1: Review of literature published since initial search (Aug 2021 – Aug 2024)

### Introduction and Methods

This appendix provides details of papers published since the initial literature search was completed in August 2021. The same methodology was followed as described in the main paper.

Using the same search criteria as detailed in Appendix 1, an additional literature search was conducted in August 2024 returning 39 results. Ten papers were excluded after title screening, nine papers were excluded after abstract screening and nine papers were excluded after full-text screening.

Data analysis was conducted independently of the original synthesis. Any themes identified in the update covering the same concept were assigned according to themes identified in the original synthesis for comparability. New themes were assigned to the most relevant of the three overarching domains from the original synthesis.

### Results

A total of eleven studies are included in this update, from which verbatim quotes, themes and findings were extracted. Reasons for exclusion at full-text screening included research focus not quality of life (Abrar et al., 2021; Bennett et al., 2021; Ceuleers et al., 2023; Eichengreen et al., 2022; Leroi et al., 2021; Littlejohn et al., 2022), inability to distinguish data from adult participants and those under 18 (Koto et al., 2022) and no reporting of qualitative data (Dhokai et al., 2023; Quimby et al., 2024).

The included papers have a combined sample size of 157 individuals with various hearing conditions. Four papers focused on the impact of hearing loss due to a specific disease or treatment, i.e., Lassa fever, osteogenesis imperfecta, chemotherapy and multidrug-resistant tuberculosis.

Data collection covered seven reported countries and one paper did not reported the nine countries where data collection took place (Goderie et al., 2023). The included countries were UK (3), South Africa (2) and Australia, Cameroon, Nepal, Nigeria, USA (all 1).

Nine of the eleven studies used semi-structured interviews, one used in-depth interviews and one used focus groups. Nine of the eleven studies used thematic analysis or a variation of thematic analysis, one used content analysis and the other did not report the type of analysis (Goderie et al., 2023).

#### Synthesis of themes

**Physical**

**Sound discrimination (pitch/clarity/localisation):** Participants who experienced gradual or sudden hearing loss reported not being able to hear certain pitches or intermittent hearing, which sometimes necessitated physical repositioning (Holman et al., 2023; Ntlhakana & Hamid, 2024; Pearson et al., 2022).

**Speech intelligibility:** Physical position and background noise were reported to affect speech intelligibility (Goderie et al., 2023; Holman et al., 2023; Lo et al., 2024; Pearson et al., 2022). People who have reduced mobility, including those with Osteogenesis Imperfecta may be particularly affected if they are not able to reposition as easily (Goderie et al., 2023).

**Mental**

**Anxiety (Worry):** Participants mentioned feelings of anxiety and worry in relation to their hearing condition, primarily in social and professional situations (de Cates et al., 2023; Holman et al., 2023; Lo et al., 2024). One participant described their anxiety when alone: “I get anxious when I’m by myself because I don’t really hear people behind me, and you get a wee bit jumpy and agitated like that sometimes as well, it makes me uncomfortable” (Holman et al., 2023).

**Depression (Sadness):** Feelings of depression or sadness are also mentioned by people with hearing conditions(de Cates et al., 2023; Goderie et al., 2023; Goodwin et al., 2024; Holman et al., 2023). De Cates et al. (2023) reported that “many participants described the feeling of sadness in relation to their symptoms with one participant commenting that his ‘general life is bad and difficult due to hearing loss”.

**Fatigue (mental tiredness):** The theme of fatigue or mental tiredness was reported in two studies (Goderie et al., 2023; Holman et al., 2023).

**Identity and Self-worth:** The theme of identity was reported in several studies; it mostly evoked negative emotions in participants such as embarrassment (Goderie et al., 2023; Holman et al., 2023; Lo et al., 2024). People’s self-worth may vary with the context in which they consider themselves, e.g., part of the hearing loss community or in hearing social circles (Holman et al., 2023). Sometimes it was a positive experience as described in Lo et al. (2024) “That visibility—that shows me what I am... I just can't imagine life not being a deaf person. I'm proud I was born deaf”.

**Fear:** Individuals with hearing loss may experience fear in certain circumstances (de Cates et al., 2023; Goderie et al., 2023; Holman et al., 2023; Lo et al., 2024). Focus group participants mentioned their concern about the difficulty of expressing themselves in emergency situations because of their hearing loss (Goderie et al., 2023).

**Social**

**Communication:** Communication needs and communication difficulties are common themes in studies of the impact of hearing on QoL (Best et al., 2024; Goderie et al., 2023; Goodwin et al., 2024; Hay-McCutcheon et al., 2023; Holman et al., 2023; Lo et al., 2024; Ntlhakana & Hamid, 2024). Difficulties communicating can have a downstream impact on many aspects of quality of life including the following themes identified.

**Social participation/Isolation:** People with hearing loss can often have challenges with social participation, particularly when communicating with people who do not have hearing loss or in noisy environments (Goderie et al., 2023; Goodwin et al., 2024; Holman et al., 2023; Lo et al., 2024; Ntlhakana & Hamid, 2024; Pearson et al., 2022; Thusi & Paken, 2022; Wonkam-Tingang et al., 2021). These experiences may cause individuals to avoid social interaction leading to feelings of isolation or loneliness (Goderie et al., 2023; Goodwin et al., 2024; Hay-McCutcheon et al., 2023; Holman et al., 2023; Lo et al., 2024).

**Relationships:** Personal and professional relationships can be impacted by hearing loss and communication needs (Holman et al., 2023; Lo et al., 2024; Ntlhakana & Hamid, 2024; Thusi & Paken, 2022). Some studies identified the importance of prior relationships and support networks for people with hearing loss, participants describe conversations with those they have close relationships with as more relaxed (Holman et al., 2023; Lo et al., 2024). Hearing loss may negatively affect relationships as described in Holman (2023): “It impacted on my relationships ... caused me to feel ignored, isolated, unsupported”.

**Stigma/Discrimination :** People with hearing conditions may experience social stigma (Best et al., 2024; de Cates et al., 2023; Holman et al., 2023; Lo et al., 2024; Wonkam-Tingang et al., 2021). Historical inaccurate associations between deafness and low intellect remain (Holman et al., 2023; Lo et al., 2024; Wonkam-Tingang et al., 2021); as evidenced in Holman et al. (2023) “They think you’re a bit daft if you ask them to repeat things”. People with hearing conditions may also face discrimination. Lo et al. (2024) reported that all participants (12) had experienced some level of stigma and discrimination due to their deafness. Discrimination was reported in professional settings as described by two participants in Lo et al. (2024): “I don't have any issues with it. In saying that if I were to go for a job interview tomorrow, to be honest, I probably would cover it. Discrimination unfortunately... It is still out there...”.

**Work/Education function:** Challenges with work or education function due to hearing conditions are reported in several studies (de Cates et al., 2023; Goderie et al., 2023; Holman et al., 2023; Lo et al., 2024; Ntlhakana & Hamid, 2024; Thusi & Paken, 2022; Wonkam-Tingang et al., 2021). Challenges may be related to finding employment, communicating with colleagues or lack of support from employers which may lead to stress (Holman et al., 2023; Lo et al., 2024; Wonkam-Tingang et al., 2021). Some participants felt excluded for work scenarios such as meetings (Holman et al., 2023; Wonkam-Tingang et al., 2021): “There is somehow discrimination compared to people who can hear. They are not invited to some meetings; they are side-lined. They think that deaf people are not equal to normal people; they are always side-lined from some meetings. They say that he is deaf, what can he do? What can he say?” (Wonkam-Tingang et al., 2021).

**Access to medical/social services:** Difficulties accessing medical care or social services were reported by several participants(Goderie et al., 2023; Goodwin et al., 2024; Lo et al., 2024; Wonkam-Tingang et al., 2021). Reasons cited were a lack of sign language translation and feeling anxious about communicating with healthcare professionals, as described below: **“**This time I didn't have somebody else to be my ears. Going to appointments alone I feel anxious because I don't know if I would be able to hear, or feel stupid, or look stupid in front of them...” (Lo et al., 2024).

**Financial issues:** Some participants had experienced a financial impact on their QoL related to their hearing condition; this was related to difficulties paying for treatment or reduced/no employment (Goodwin et al., 2024; Hay-McCutcheon et al., 2023; Thusi & Paken, 2022).

**Coping strategies/mechanisms:** Coping strategies are often a part of daily life for people with hearing conditions; these involve situating themselves so they can see the faces of communication partners or turning to their better hearing side and avoiding noisy places (de Cates et al., 2023; Holman et al., 2023; Lo et al., 2024).

#### Quality Assessment

The quality of the included papers was assessed using the CASP checklist. Overall, the reporting of the studies was adequate and relevant considerations were undertaken. In 6 of the 11 papers, it was not possible to tell whether the relationship between the researcher and participant had been adequately considered; this was also observed in the papers included in the initial literature search.

### Discussion

Overall, the themes identified in the update were similar to those identified in the initial qualitative synthesis. Themes from the initial qualitative synthesis not identified in the update are physical fatigue, listening effort, confidence and independence. Themes in the physical domain of the initial analysis were identified with less specificity, and so were reported as ‘Sound discrimination’. If the data from the initial search and update had been analysed together, it’s likely that these themes would have been identified.

The additional themes of access to medical/social services and financial issues were included in this update. Both of these themes have been explored as part of the hearing literature more generally, and may have downstream implications on health outcomes (Huddle et al., 2017; Michael et al., 2019; Shukla et al., 2019; Weerapol & Leelakanok, 2024). Additionally, these themes aren’t fully aligned with the social category, together with work function, a socioeconomic category maybe more appropriate.

The updated synthesis includes a greater proportion of studies conducted in a low- or middle-income country (LMIC) (Best et al., 2024; de Cates et al., 2023; Ntlhakana & Hamid, 2024; Thusi & Paken, 2022; Wonkam-Tingang et al., 2021); which was identified as a limitation of the original synthesis. The additional themes could be partly attributed to them being more apparent in LMICs. However, they could also be experienced by individuals with hearing conditions living in high-income countries.

### Conclusion

Hearing conditions have a significant impact on various domains of quality of life, often impacting physical, mental and social aspects of individuals’ lives. The themes identified in this update broadly align with previously published literature on the impact of quality of life in people with hearing conditions as discussed in the main article.

### References

Abrar, R., Bruce, I. A., O’Driscoll, M., Freeman, S., de Estibariz, U. M., & Stapleton, E. (2021). Impact on patients of the coronovirus disease 2019 pandemic and postponement of cochlear implant surgery: A qualitative study. *The Journal of Laryngology and Otology*, *135*(10), 918–925. https://doi.org/10.1017/S002221512100219X

Bennett, R. J., Donaldson, S., Kelsall-Foreman, I., Meyer, C., Pachana, N. A., Saulsman, L., Eikelboom, R. H., & Bucks, R. S. (2021). Addressing Emotional and Psychological Problems Associated With Hearing Loss: Perspective of Consumer and Community Representatives. *American Journal of Audiology*, *30*(4), 1130–1138. https://doi.org/10.1044/2021_AJA-21-00093

Best, K. C., Ameh, E., Weldon, C., Shwe, D., Maigari, I. M., Turaki, I., Ma’an, N. D., Yilgwan, C. S., Makishima, T., Weaver, S., Paessler, S., & Shehu, N. Y. (2024). Double stigma: A cross-sectional study of Lassa patients with hearing loss in North Central Nigeria. *Frontiers in Public Health*, *12*, 1395939. https://doi.org/10.3389/fpubh.2024.1395939

Ceuleers, D., Baudonck, N., Keppler, H., Kestens, K., Dhooge, I., & Degeest, S. (2023). Development of the hearing-related quality of life questionnaire for auditory-visual, cognitive and psychosocial functioning (hAVICOP). *Journal of Communication Disorders*, *101*, 106291. https://doi.org/10.1016/j.jcomdis.2022.106291

de Cates, C., Jashek-Ahmed, F., Bohara, R. B., Salter, C., & Youngs, R. (2023). How chronic ear disease affects quality of life: A qualitative research study in Nepal. *The Journal of Laryngology and Otology*, *137*(4), 390–397. https://doi.org/10.1017/S0022215122001050

Dhokai, N., Matto, H., Ihara, E. S., Tompkins, C. J., Caswell, S. V., Cortes, N., Davis, R., Coogan, S. M., Fauntroy, V. N., Glass, E., Lee, J. M., Baraniecki-Zwil, G., & Ambegaonkar, J. P. (2023). Community arts engagement supports perceptions of personal growth in older adults. *Journal of Aging Studies*, *66*, 101142. https://doi.org/10.1016/j.jaging.2023.101142

Eichengreen, A., Zaidman-Zait, A., Most, T., & Golik, G. (2022). Resilience from childhood to young adulthood: Retrospective perspectives of deaf and hard of hearing people who studied in regular schools. *Psychology & Health*, *37*(3), 331–349. https://doi.org/10.1080/08870446.2021.1905161

Goderie, T., Hendricks, S., Cocchi, C., Maroger, I. D., Mekking, D., Mosnier, I., Musacchio, A., Vernick, D., & Smits, C. (2023). The International Standard Set of Outcome Measures for the Assessment of Hearing in People with Osteogenesis Imperfecta. *Otology & Neurotology : Official Publication of the American Otological Society, American Neurotology Society [and] European Academy of Otology and Neurotology*, *44*(7), e449–e455. https://doi.org/10.1097/MAO.0000000000003921

Goodwin, M. V., Hogervorst, E., & Maidment, D. W. (2024). A qualitative study assessing the barriers and facilitators to physical activity in adults with hearing loss. *British Journal of Health Psychology*, *29*(1), 95–111. https://doi.org/10.1111/bjhp.12689

Hay-McCutcheon, M. J., Brothers, E. B., & Allen, R. S. (2023). An Assessment of Hearing Health Care Needs in Rural West Central and South Alabama. *American Journal of Audiology*, *32*(3), 487–499. https://doi.org/10.1044/2023_AJA-22-00177

Holman, J. A., Ali, Y. H. K., & Naylor, G. (2023). A qualitative investigation of the hearing and hearing-aid related emotional states experienced by adults with hearing loss. *International Journal of Audiology*, *62*(10), 973–982. https://doi.org/10.1080/14992027.2022.2111373

Huddle, M. G., Goman, A. M., Kernizan, F. C., Foley, D. M., Price, C., Frick, K. D., & Lin, F. R. (2017). The Economic Impact of Adult Hearing Loss: A Systematic Review. *JAMA Otolaryngology–Head & Neck Surgery*, *143*(10), 1040–1048. https://doi.org/10.1001/jamaoto.2017.1243

Koto, Y., Narita, A., Noto, S., Ono, M., Hamada, A. L., & Sakai, N. (2022). Qualitative analysis of patient interviews on the burden of neuronopathic Gaucher disease in Japan. *Orphanet Journal of Rare Diseases*, *17*(1), 280. https://doi.org/10.1186/s13023-022-02429-z

Leroi, I., Wolski, L., Charalambous, A. P., Constantinidou, F., Renaud, D., Dawes, P., Hann, M., Himmelsbach, I., Miah, J., Payne, M., Simkin, Z., Thodi, C., Yeung, W. K., & Yohannes, A. M. (2021). Support care needs of people with hearing and vision impairment in dementia: A European cross-national perspective. *Disability and Rehabilitation*, 1–13. https://doi.org/10.1080/09638288.2021.1923071

Littlejohn, J., Bowen, M., Constantinidou, F., Dawes, P., Dickinson, C., Heyn, P., Hooper, E., Hopper, T., Hubbard, I., Langenbahn, D., Nieman, C. L., Rajagopal, M., Thodi, C., Weinstein, B., Wittich, W., & Leroi, I. (2022). International Practice Recommendations for the Recognition and Management of Hearing and Vision Impairment in People with Dementia. *Gerontology*, *68*(2), 121–135. https://doi.org/10.1159/000515892

Lo, C. Y., Clay-Williams, R., Elks, B., Warren, C., & Rapport, F. (2024). The (in)visibility of deafness: Identity, stigma, quality of life and the potential role of totally implantable cochlear implants. *Health Expectations : An International Journal of Public Participation in Health Care and Health Policy*, *27*(3), e14060. https://doi.org/10.1111/hex.14060

Michael, R., Attias, J., & Raveh, E. (2019). Perceived Quality of Life Among Adults With Hearing Loss: Relationships With Amplification Device and Financial Well-Being. *Rehabilitation Counseling Bulletin*, *62*(4), 234–242. https://doi.org/10.1177/0034355217738717

Ntlhakana, L., & Hamid, S. (2024). Exploring quality of life post sudden onset hearing loss: A convergent parallel approach. *The South African Journal of Communication Disorders = Die Suid-Afrikaanse Tydskrif Vir Kommunikasieafwykings*, *71*(1), e1–e7. https://doi.org/10.4102/sajcd.v71i1.990

Pearson, S. E., Caimino, C., Shabbir, M., & Baguley, D. M. (2022). The impact of chemotherapy-induced inner ear damage on quality of life in cancer survivors: A qualitative study. *Journal of Cancer Survivorship : Research and Practice*, *16*(5), 976–987. https://doi.org/10.1007/s11764-021-01089-5

Quimby, A. E., Brant, J. A., Staab, J. P., & Ruckenstein, M. J. (2024). Development and Initial Validation of a Meniere’s Disease Quality of Life Instrument: The MenQOL. *The Laryngoscope*. https://doi.org/10.1002/lary.31478

Shukla, A., Nieman, C. L., Price, C., Harper, M., Lin, F. R., & Reed, N. S. (2019). Impact of Hearing Loss on Patient–Provider Communication Among Hospitalized Patients: A Systematic Review. *American Journal of Medical Quality*, *34*(3), 284–292. https://doi.org/10.1177/1062860618798926

Thusi, A. B., & Paken, J. (2022). The lived experiences and psychosocial impact of hearing loss on the quality of life of adults with Multidrug-Resistant Tuberculosis. *The South African Journal of Communication Disorders = Die Suid-Afrikaanse Tydskrif Vir Kommunikasieafwykings*, *69*(1), e1–e13. https://doi.org/10.4102/sajcd.v69i1.823

Weerapol, N., & Leelakanok, N. (2024). Communication between healthcare professionals and patients with hearing loss: A systematic review and meta-analysis. *American Journal of Health-System Pharmacy*, *81*(12), 521–530. https://doi.org/10.1093/ajhp/zxae045

Wonkam-Tingang, E., Kengne Kamga, K., Adadey, S. M., Nguefack, S., De Kock, C., Munung, N. S., & Wonkam, A. (2021). Knowledge and Challenges Associated With Hearing Impairment in Affected Individuals From Cameroon (Sub-Saharan Africa). *Frontiers in Rehabilitation Sciences*, *2*, 726761. https://doi.org/10.3389/fresc.2021.726761

Table S1: Summary of the 11 articles included in the update

| **Reference (Author, year)** | **Country/ countries of data collection** | **Participant sample (type of hearing problem)** | **No. of participants** | **No. of Data points, e.g., focus groups, interviews)** | **Method of data collection** | **Type of analysis** | **Research focus** **(e.g., patient groups or outcomes of interest)** |
| --- | --- | --- | --- | --- | --- | --- | --- |
| Best et al., 2024 | Nigeria | Lassa patients with hearing loss | 15 | Each participant was interviewed once individually. | In-depth interview | Thematic analysis | Stigma in Lassa patients with hearing loss |
| de Cates et al., 2023 | Nepal | Chronic ear disease | 20 | Each participant was interviewed once individually. | Semi-structured interviews | Thematic content analysis | QoL due to chronic ear disease |
| Goderie et al., 2023 | NR (Nine countries) | Hearing loss and Osteogenesis Imperfecta (OI) | 22 | 3 focus groups | Focus Group | NR | Outcome measure for assessment of hearing in OI |
| Goodwin et al., 2024 | UK | Hearing loss | 10 | Each participant was interviewed once individually. | Semi-structured interviews | Thematic analysis | Barriers and facilitators to physical activity in adults with HL |
| Hay-McCutcheon et al., 2023 | USA | Hearing loss | 26 | Each participant was interviewed once individually. | Semi-structured interviews | Content analysis | Hearing health care needs in rural Alabama |
| Holman et al., 2023 | UK | Hearing loss | 17 | Each participant was interviewed once individually | Semi-structured interviews | Thematic analysis | Hearing and hearing-aid related emotional states |
| Lo et al., 2024 | Australia | Cochlear impact users | 12 | Each participant was interviewed once individually | Semi-structured interviews | Thematic analysis | Identity, stigma, QoL in CI users |
| Ntlhakana & Hamid, 2024 | South Africa | Sudden Onset Hearing Loss (SOHL) | 2 | Each participant was interviewed once individually | Semi-structured interviews | Descriptive and thematic analysis | QoL in people with SOHL |
| Pearson, 2022 | UK | Chemotherapy-induced ear damage | 20 | Each participant was interviewed once individually | Semi-structured interviews | Thematic analysis | QoL in cancer survivors with ototoxicity |
| Thusi & Paken, 2022 | South Africa | Hearing loss in adult with Multidrug-resistant Tuberculosis | 10 | Each participant was interviewed once individually | Semi-structured interviews | Thematic analysis | Lived experiences, psychosocial impact and QoL due to HL |
| Wonkam-Tingang et al., 2022 | Cameroon | Hearing impairment | 3 | Each participant was interviewed once individually | Semi-structured interviews | Thematic analysis | Hearing Impairment in Cameroon |

NR= Not Reporte

# **Supplementary Material 2: PubMed Search Strategy**

**Table S2. PubMed Search strategy for identification of relevant articles for the synthesis**

| **Search** | **Query** |
| --- | --- |
| #1 | (("Qualitative Research"[Mesh] OR "Focus Groups"[Mesh] OR qualitative research*[tiab] OR qualitative method*[tiab] OR qualitative approach*[tiab] OR focus group*[tiab] OR (("Interview" [Publication Type] OR "Interviews as Topic"[Mesh] OR interview*[tiab]) AND (“semi-structured”[tiab] OR semistructured[tiab] OR unstructured[tiab] OR structured[tiab] OR informal*[tiab] OR “in-depth”[tiab] OR indepth[tiab] OR guide[tiab] OR guides[tiab] OR qualitative[tiab])))) |
| #2 | (“Hearing loss”[Mesh] OR “Hearing disorders”[Mesh] OR Hearing impair*[tiab] OR “Deafness”[Mesh] OR “Tinnitus”[Mesh] OR Hearing aid[tiab] OR Cochlear implant[tiab] OR Meniere’s disease[tiab]) |
| #3 | ("Quality of Life"[Mesh] OR "Happiness"[Mesh] OR "Personal Satisfaction"[Mesh] OR life qualit*[tiab] OR "quality of life"[tiab] OR qol[tiab] OR happiness*[tiab] OR life satisf*[tiab] OR well being[tiab] OR wellbeing[tiab]) |
| #4 | #1 AND #2 AND #3 |

# **Supplementary Material 3: CASP Checklist**

**Table S3. CASP checklist reporting**

Note that question 11 of the revised tool (question 10 of the original tool) is open-ended and is therefore not included. For the complete original checklist see (25), and for the revised tool, see (28).

|  | Study aims clearly stated | Appropriate Methods | Justification of methods | Clear and coherent theoretical underpinnings | Recruitment strategy given | Data collection described | Researcher bias discussed | Study ethics described | Data analysis described | Results described |
| --- | --- | --- | --- | --- | --- | --- | --- | --- | --- | --- |
| Barlow et al.(44) | Yes | Yes | Yes | Yes | Yes | Yes | Yes | Can't tell | Yes | Yes |
| Bennion et al. (40) | Yes | Yes | Yes | Yes | Yes | Yes | Can't tell | Yes | Yes | Yes |
| Brooker et al. (35) | Yes | Yes | Yes | Yes | Yes | Yes | Yes | Yes | Yes | Yes |
| Buhagiar & Lutman (45) | Yes | Yes | Yes | Can’t tell | Can't tell | Yes | Can't tell | Can't tell | No | No |
| Davis et al. (34) | Yes | Yes | Yes | Yes | Yes | Yes | Yes | Yes | Yes | Yes |
| Dixon et al. (36) | Yes | Yes | Yes | Yes | Yes | Yes | Yes | Yes | Yes | Yes |
| Duchesne et al. (33) | Yes | Yes | Yes | Yes | Yes | Yes | Yes | Can't tell | Yes | Yes |
| Hughes et al. (46) | Yes | Yes | Yes | Yes | Yes | Yes | Yes | Yes | Yes | Yes |
| Ingram et al. (43) | Yes | Yes | Yes | Can’t tell | Yes | Yes | Can't tell | Yes | Yes | Yes |
| Jeffs et al. (39) | Yes | Yes | Yes | Yes | Yes | Yes | Yes | Yes | Yes | Yes |
| Kushalnagar et al. (41) | Yes | Yes | Yes | Yes | Can't tell | Yes | No | Can't tell | Yes | Yes |
| Lucas et al. (37) | Yes | Yes | Yes | Yes | Yes | Yes | Yes | Yes | Yes | Yes |
| McAbee et al. (53) | Yes | Yes | Yes | Yes | Yes | Yes | Yes | Can't tell | Yes | Yes |
| McRackan et al. (38) | Yes | Yes | Yes | Yes | Yes | Yes | Yes | Yes | Yes | Yes |
| Mealings et al. (42) | Yes | Yes | Yes | Yes | Yes | Yes | Can't tell | Yes | Can't tell | Yes |
| Ng et al. (51) | Yes | Yes | Yes | Yes | Yes | Yes | Can't tell | Yes | Can't tell | Yes |
| Powell et al. (52) | Yes | Yes | Yes | Yes | Yes | Yes | Can’t tell | Yes | Yes | Yes |
| Pryce & Chilvers (56) | Yes | Yes | Yes | Yes | Yes | Yes | Can't tell | Yes | Yes | Yes |
| Punch et al. (54) | Yes | Yes | Yes | Yes | Yes | Yes | Yes | Yes | Yes | Yes |
| Rapport et al. (48) | Yes | Yes | Yes | Yes | Yes | Yes | Can't tell | Yes | Yes | Yes |
| Vieira et al. (32) | Yes | Yes | Yes | Yes | Yes | Yes | Can't tell | Yes | Yes | Yes |
| Yuan et al. (47) | Yes | Yes | Yes | Yes | Yes | Yes | Yes | Yes | Yes | Yes |
